# Variables associated with interprofessional collaboration: a comparison between primary healthcare and specialized mental health teams

**DOI:** 10.1186/s12875-019-1076-7

**Published:** 2020-01-08

**Authors:** Nicolas Ndibu Muntu Keba Kebe, François Chiocchio, Jean-Marie Bamvita, Marie-Josée Fleury

**Affiliations:** 1 Department of Management, Evaluation and Health Policy, Université de Montréal, School of Public Health, 7101 Parc Avenue, Montreal, Quebec H3N 1X9 Canada; 2 Telfer School of Management, University of Ottawa, 55 Laurier Avenue East, Ottawa, Ontario K1N 6N5 Canada; 3 Douglas Hospital Research Centre, Douglas Mental Health University Institute, 6875 LaSalle Boulevard, Montreal, Quebec H4H 1R3 Canada; 4 Department of Psychiatry, McGill University; Douglas Hospital Research Centre, Douglas Mental Health University Institute, 6875 LaSalle Boulevard, Montreal, Quebec H4H 1R3 Canada

**Keywords:** Interprofessional collaboration, Mental health teams, Variables associated, Primary care teams, Specialized services teams

## Abstract

**Background:**

This study has two aims: first, to identify variables associated with interprofessional collaboration (IPC) among a total of 315 Quebec mental health (MH) professionals working in MH primary care teams (PCTs, *N* = 101) or in specialized service teams (SSTs, *N* = 214); and second, to compare *IPC associated variables* in MH-PCTs vs MH-SSTs.

**Methods:**

A large number of variables acknowledged as strongly related to IPC in the literature were tested. Multivariate regression models were performed on MH-PCTs and MH-SSTs respectively.

**Results:**

Results showed that knowledge integration, team climate and multifocal identification were independently and positively associated with IPC in both MH-PCTs and MH-SSTs. By contrast, knowledge sharing was positively associated with IPC in MH-PCTs only, and organizational support positively associated with IPC in MH-SSTs. Finally, one variable (age) was significantly and negatively associated with IPC in SSTs.

**Conclusions:**

Improving IPC and making MH teams more successful require the development and implementation of differentiated professional skills in MH-PCTs and MH-SSTs by care managers depending upon the level of care required (primary or specialized). Training is also needed for the promotion of interdisciplinary values and improvement of interprofessional knowledge regarding IPC.

## Background

Interprofessional collaboration (IPC) is defined as a process by which professionals from multiple disciplines share roles and tasks in order to respond in a coherent and integrated way to the needs of patients, their loved ones and the community [1, 2]. IPC has benefits for patients, health professionals, healthcare organizations and health systems [3, 4]. Studies have shown that good IPC reduces healthcare costs and expenditures, enhances quality of care and increases job satisfaction, improving staff retention and patient outcomes [5, 6]. IPC responds to the shortage of financial, human, and technical resources by providing solutions that increase the effectiveness and efficiency of health services, while better responding to the complex needs of patients with chronic conditions [1, 7].

Despite abundant evidence for the positive effects of IPC, studies have shown that the uptake of IPC in organizations remains weak; IPC is also inadequately practiced in healthcare teams [8, 9]. Inadequate IPC has been associated with medication errors, patient safety problems, team conflict and patient mortality [10, 11]. Thus, there is a great need for research identifying variables associated with IPC, particularly in mental health (MH) primary care teams (PCTs) but also in MH specialized service teams (SSTs) [12, 13]. MH service reforms have been introduced [14, 15] with the aim of improving interdisciplinary teamwork [16–18]. Reforms have focused on improving interdisciplinary collaboration among professionals and team consolidation within MH-PCTs and MH-SSTs, but also on integrating primary and specialized care teams to provide better service for patients [19, 20]. MH-PCTs using more limited expertise mainly treat patients with common mental disorders [21], making use of a restricted number of treatment sessions and brief interventions [22, 23]. This may include diagnosis and treatment of mild to moderate depression and anxiety, the provision of a range of short-term biopsychosocial treatment interventions, and advice on referral options [24, 25]. Conversely, MH-SSTs mostly treat patients with serious mental disorders or co-occurring mental disorders and substance use disorders, requiring specialist intervention with more intensive sessions and a longer time frame. Increasing evidence suggests the co-occurrence of substance use disorders and psychiatric illness such as schizophrenia and bipolar disorder, rendering treatment more difficult and resulting in greater use of diversified healthcare services [26]. In this context, the call for complex care involving multiple professionals from specialized services, and the need for professional collaboration is higher in SSTs than in primary healthcare [27, 28], where most previous research identifying variables related to IPC in MH has occurred [8, 13]. The identification and comparison of variables associated with IPC among professionals working in MH-PCTs and MH-SSTs may provide a better understanding regarding the nature of collaboration among MH professionals in these different service settings in terms of maximizing IPC and making MH teams more successful.

To this end, a classification of variables that may influence IPC was constructed based on the Bronstein model [29], and on studies by San Martin-Rodriguez, Beaulieu, D’Amour, & Ferrada-Videla [30] and Mulvale et al. [13] considering previous empirical research and data collection for this study. All variables recognized in the scientific literature in the health field as strongly associated with IPC were considered, and were categorized within this model. The model consisted of four conceptual blocks including individual, interactional, structural and professional role characteristics*.* Individual characteristics include demographic variables (e.g., gender, age) and personal attributes [14]; while interactional characteristics refer to those taking place among team members [30]. Structural or organizational characteristics include factors beyond the control of any individual team member; while professional role characteristics include self-identity in individual professional practice [29].

Concerning individual characteristics, age, seniority on the team and belief in the benefits of interdisciplinary collaboration have been identified in prior studies as positively associated with IPC [31–33]*.* In addition, Pounder & Coleman [34] found that women tend to work more collaboratively than men in teams.

Regarding interactional characteristics, previous research has identified knowledge sharing and knowledge integration as positively related to IPC [13, 30, 33, 35]. Knowledge sharing is defined as the act of providing or transferring knowledge to others [36]; while knowledge integration refers to the ability of team members to bring together knowledge from different disciplines to meet the needs of people with chronic and/or complex health conditions for which no single health professional has the requisite expertise [37]. Previous research also found that participation in decision-making, affective commitment toward the team, mutual trust and team climate were positively related to IPC [13, 14, 35]. Participation in decision-making refers to the discussions held among professionals working in a team that result in decisions around patient care based on consensus; while affective commitment toward the team is defined as the professional‘s psychological and emotional attachment to his/her team [38]. Any worker given more responsibility and decision-making power over his/her job will produce higher quality work and achieve a higher level of job performance and satisfaction [13, 39, 40]. Previous research has also identified mutual trust and *team climate* as other interactional characteristics positively related to IPC [13, 30, 33, 35]. Better team climate contributed to mutual respect and cohesion in teams [41]. By contrast, research identified employee conflict as negatively associated with IPC [13, 42]. The confluence of several disciplines in managing complex or chronic cases, and diversity among individual professionals, tended to increase the potential for conflict in MH teams. Team conflict may hinder decision-making, team functioning and effectiveness while negatively impacting patient care and job performance [43]. Moreover, team autonomy, meaning work groups allowed sufficient organizational latitude to establish their own internal goals and work practices, is another interactional characteristic related to IPC that has produced mixed results, as reported in various studies: one finding positive associations between team autonomy and IPC [44], while another revealing that greater team autonomy may have adverse effects for IPC and team effectiveness [45]*.* Yet the benefits of team autonomy are clearly established in terms of a better exchange of skills, creativity, cohesion among team members and more effective group decision-making [46, 47], identifying team autonomy as a highly sought-after quality in the MH domain.

Regarding structural characteristics that may influence IPC, positive relationships between organizational support and IPC have been identified in previous research [3, 33, 48]. Organizational support reflects employee perceptions of the extent to which an organization values its contribution and the degree of focus on organizational well-being; organizational support also includes logistical, administrative, and clinical supervision, and the development of training protocols or tools to assist employees in their work [49, 50]. Results showed the highly positive impact of organizational support in terms of team performance [51] and reduced staff turnover [52]. Team size is another structural characteristics studied by many researchers in relation to IPC with mixed results [3, 13]. One study found that members working in larger teams were less effective, less involved in decision-making processes and in IPC [3]; whereas another reported that small teams were highly dependent on the individual skills, abilities and experience of their members for meeting the biopsychosocial or multidimensional needs of patients [13]*.*

Concerning professional role characteristics, type of profession, team identification and professional identification have been frequently studied in relation to IPC. While the cultures and values of particular healthcare professions create challenges for effective teamwork, reducing IPC effectiveness [53, 54], other healthcare professions transmit values, beliefs and behaviours that promote IPC [55]. Moreover, studies have demonstrated that team identification creates a sense of unity and solidarity among team members, improving IPC [56, 57]. By contrast, professional identification deriving from professional commitment and specific related practices was negatively related to IPC [58, 59]. However, to the best of our knowledge, no studies have measured the impact of both team identification and professional identification, namely multifocal identification, on IPC. More concretely, multifocal identification refers to the fact that any team member identifies simultaneously with the team (team identification) and with his/her profession (professional identification) [60–62]*.*

Many studies have tested variables for their influence on IPC. However, research has yet to compare variables associated with IPC among professionals working in MH-PCTs vs MH-SSTs. Based on the conceptual framework developed by Bronstein et al. [29], and further enhanced by the work of San Martín-Rodríguez et al. [29] and Mulvale et al. [13], this study thus aimed to identify variables associated with IPC, comparing MH-PCTs and MH-SSTs. As these two types of teams differ in terms of their activities, composition, client base, roles and functions, it was hypothesized that different variables related to MH-PCTs or MH-SSTs may influence IPC.

## Methods

### Study design and sample

This study emanated from a larger evaluation of local health service networks (LHSNs) implemented under the 2005–2015 MH care reform [63, 64] in Quebec (Canada). As part of a global reform of the Quebec healthcare system [17], general hospitals, local community health centers, and nursing homes were merged to create 95 health and social service centers (HSSCs), mandated to oversee health service organization in their respective LHSNs, and to coordinate the various health service providers (e.g., psychiatric hospitals, community-based organizations, and medical clinics). The reform [17] required that at least one MH-PCT be established within each HSSC to treat patients with common mental disorders and provide follow-up services to stabilized service users with severe mental disorders. MH-PCT for the adult population integrated local teams such as biopsychosocial teams, and MH professionals (e.g. social workers, educators) based on roughly six psychosocial clinicians and 0.5 general practitioners per 100,000 inhabitants in the territory. MH-SST teams operated within hospital MH services (e.g. day hospital units, outpatient clinics, assertive community treatment).

The study included MH professionals from four Quebec LHSNs selected in consultation with an advisory committee composed of key decision makers in the Quebec MH care system [18]. Three LHSNs were served by a psychiatric hospital: two in the province’s largest city and one in the capital. The fourth, located in a semi-urban area, relied on the services of a psychiatric department in a general hospital. Population areas ranged from 135,000 inhabitants in the semi-urban area to 300,000 served by the LHSN in the capital city.

To be eligible for the study, MH professionals had to work in one of the four selected LHSNs as members of a MH-PCT or MH-SST, and on teams composed of three or more MH professionals from at least two disciplines (e.g., psychologist, nurse, and social worker). A list of all MH professionals working in the teams that met these requirements was provided by the advisory committee. All potential study participants were contacted by email or telephone and invited to the study. A psychiatric research ethics board approved the multisite study protocol.

### Data collection and variables

**Fig. 1:**
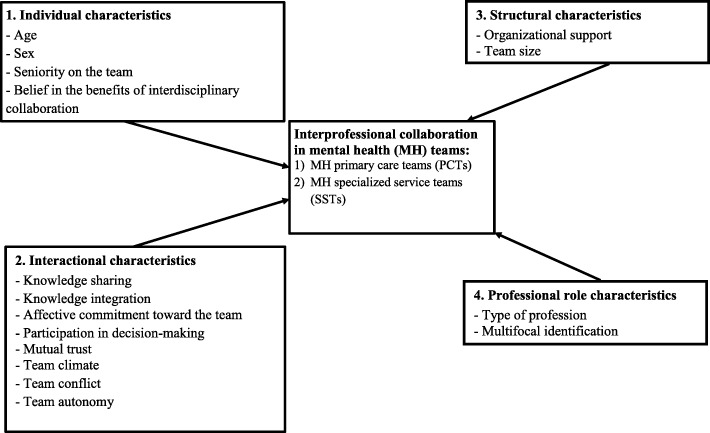
Conceptual framework

A total of 466 MH professionals working in MH teams (154 in PCTs and 312 in SSTs) across the four LHSNs were invited to the study. Data collection involved the mailing of self-administered questionnaires to study participants, who completed and returned them between May 2013 and November 2014. To optimize the response rate, several recruitment strategies were conducted, including invitations by email and telephone, and information sessions held with MH professionals in PCTs and SSTs, and with their managers, who further assisted with recruitment. The questionnaire, which took approximately 45 min to complete, included sociodemographic information and questions related to diverse aspects of teamwork using standardized scales. Figure 1 presents the conceptual framework for the study based on the interdisciplinary collaborative framework described above. The framework describes the dependent variable and independent variables included in the study, considering previous empirical research and data collection for this study.

Table 1 describes the instruments used in the study to measure variables in the conceptual framework, including the Cronbach’s alpha coefficients for each instrument from the original version (the English version for most), the validated French (or English) version and the study version used. The change in the Likert scales for some instruments used in the study should not reduce their reliability, validity or discriminating power [65, 66]. IPC, the dependent variable, was measured using the Team Collaboration Questionnaire by Chiocchio, Grenier, O’Neill, Savaria and Willms [67], (Cronbach α coefficient between 0.91 and 0.92) based on a 7-point Likert scale (1 = completely disagree, 7 = completely agree). There were 14 items divided into four sub-dimensions: team communication (5 items), synchronization (3 items), explicit coordination (3 items) and implicit coordination (3 items). Independent variables were organized according to the four blocks of the conceptual framework: 1) Individual characteristics (4 variables) 2) Interactional characteristics (8 variables), 3) Structural characteristics (2 variables), and 4) Professional role characteristics (2 variables). All variables related to Interactional characteristics and one variable on Individual characteristics (belief in the benefits of interdisciplinary collaboration) were measured with validated instruments using 7-point Likert scales, while a single variable on Structural characteristics (organizational support), and another on Professional role characteristics (multifocal identification) also used instruments measured with 7-point Likert scales. One of the Professional role characteristics variables, type of profession, was further categorized in terms of medical professions (e.g. specialist, general practitioner, nurse, pharmacist), psychosocial professions (e.g. social worker, psychologist), and general professions (e.g. technician, clerk).

**Table 1:** Variables and instruments used in the study

Blocks of variables		Variables	Instruments
1) Independent variables (IV):			
Individual Characteristics	1	Age	Research team socio-demographic questionnaire.
	2	Gender	Research team socio-demographic questionnaire.
	3	Belief in the benefits of interdisciplinary collaboration	Instrument designed by Sicotte et al. (2002) [1], composed of five items; 5 likert scale; Cronbach alpha (α): 0.92; (Original version in French). Global internal consistency of the version for this study; 7 likert-scale; α: 0.92.
	4	Seniority in the team	Research team socio-demographic questionnaire.
Interactional Characteristics	5	Knowledge sharing (intention to share knowledge)	Instrument designed by Bock, Zmud, Kim and Lee (2005) [2], composed of five items;. 5 likert scales; α: 0.93; (Original version). Global internal consistency of the first French version: N.A. Global internal consistency of the French version for this study; 7-likert scale: α:0.86.
	6	Knowledge integration	Instrument designed by Song and Xies (2000) [3], composed of nine items; 11 likert-scale; α: N.A.; (Original version). Global internal consistency of the first French Version: N.A. Global internal consistency of the French version for this study; 7-likert scale; α:0.95.
	7	Affective commitment toward the team	Instrument designed by Allen and Meyer (1990) [4], composed of four items; 7 likert-scale; α: 0.86–0.92; (Original version). Global internal consistency of the first French Version: N.A. Global internal consistency of the French version for this study; 7-likert scale; α:0.91.
	8	Participation in decision-making	Instrument designed by Campion, Medsker and Higgs (1993) [5], composed of three items; 5 likert-scale;. α: 0.88; (Original version). Global internal consistency of the first French version [6]; α: 0.80. Global internal consistency of the French version for this study; 7-likert scale; α:0.90.
	9	Mutual trust	Instrument designed by Simons and Peterson (2000) [7], composed of four items;5 likert-scale; α: 0.89. (Original version). Global internal consistency of the first French version [8];α:0.90. Global internal consistency of the French version for this study; 7-likert scale; α:0.92.
	10	Team climate	Instrument designed by Anderson and West (1998) [9], composed of nineteen items (4 dimensions); 5 likert-scale α: 0.84–0.92; (Original version). Global internal consistency of the First french version [10];: 0.88–0.93. Global internal consistency of the French version for this study; 7-likert scale; (sum of the 4 dimensions) α: 0.84–0.93.
	11	Team conflict	Instrument designed by Jehn and Mannix (1991) [11], composed of nine-items (3 dimensions); 5 likert-scale; α: 0.93–0.94; (Original version). Global internal consistency of the first French version [6]: α: 0.75–0.93 Global internal consistency of the French version for this study; 7-likert scale; (sum of the 4 dimensions) α: 0.84–0.91.
	12	Team autonomy	Instrument designed by Campion, Medsker and Higgs (1993) [5], composed of three-items; 5 likert-scale; α: 0.76; (Original version). Global internal consistency of the first French version [6]: α: 0.67 Global internal consistency of the French version for this study: 7-likert scale; α:0.81.
Structural Characteristics	13	Organizational support	Instrument designed by Spreitzer (1996) [12], composed of four-items. α: N.A.; (Original version). Global internal consistency of the first French version [13]; α::0.85 French version for this study; 7-likert scale: α:0.84.
	14	Team size	Research team socio-demographic questionnaire.
Professional Role Characteristics	15	Type of profession	Research team socio-demographic questionnaire.
	16	Multifocal identification	Instrument designed by Christ, Van Dick, Wagner, and Stellmacher (2003) [14], composed of 7 items;. 6 likert-scale; Cronbach alpha (α): 0.79; (Original version) Global internal consistency of the first French version [15]; α::0.69 French version for this study; 7—likert-scale: α:N.A.
2) Dependent variable (DV):		Interprofessional collaboration (IPC)	Instrument designed by Chiocchio, Grenier, O’Neil, Savaria and Willms (2012) [16], composed of a fourteen-items in four sub-dimensions: communication (5 items); synchronization (3 items), explicit coordination (3 items); implicit coordination (3 items); 7 likert-scale; α: 0.91–0.92; (Original version in French). Global internal consistency of the version for this study α:0.94:

### Analyses

After scrutinizing the database, no outliers were found, and few missing values (less than 5%), which were replaced by the means. Univariate, bivariate and multivariate analyses were performed for both IPC in primary care (MH-PCTs; *N* = 101) and IPC in specialized care (MH-SSTs; *N* = 214). Univariate analyses included frequency distributions (numbers and percentages) for categorical variables, and central tendency measurements (means, standard deviations) for continuous variables. Linear regressions were conducted for bivariate analyses to assess associations between each independent variable and the dependent variable. For the bivariate analyses, the alpha value was set at 0.10 (which is less restrictive than 0.05), as some associations identified as not significant in the bivariate analysis, if considered with an alpha value at 0.05, may become significant when tested against other variables in the multiple regressions. Variables significantly associated with each dependent variable in the bivariate analyses were then used to build the two multiple linear regression models (PCTs, SSTs) using the Backward elimination technique, with alpha set at 0.05. The total variance explained (adjusted *R*^2^) and goodness of fit (*F*-test and *p* value) were calculated for the two multiple regression models.

## Results

Three hundred fifteen (315) MH professionals working in PCTs (*N* = 101) or SSTs (*N* = 214) participated in the study, for a 67.6% response rate. With regard to healthcare services, 32.1% of MH professionals worked in MH-PCTs and 67.9% in MH-SSTs. Most MH professionals were female: 70.2% in the total sample, 78.2% in MH-PCTs, and 65.4% in MH-SSTs. No significant differences were found between participants and non-participants with respect to distributions for team type [χ2 (1, *N* = 466) =0.79; *p* = 0.68] and gender [χ 2 (1, N = 466) =0.03; *p* = 0.87]. The mean age of study participants was 43 years for the total sample, 41.7 years in MH-PCTs, and 44.1 years in MH-SSTs. Mean seniority on the teams was 3 years for the total sample, 2.25 years in MH-PCTs, and 3.44 in MH-SSTs. Most participants in both the total sample and the two subsamples were psychosocial professionals (77.2% in MH-PCTs vs. 44.4% in MH-SSTs); while the rest were medical professionals (15.8% in MH-PCTs vs. 43.5% in MH-SSTs) or general professionals (7.9% in MH-PCTs vs. 12.1% in MH-SSTs). IPC had a mean score of 8.022 (SD = 3.751) for PCTs, and 19.931 (SD = 3.747) for SSTs. Participant characteristics are presented in Table 2, along with significant variables from the bivariate analyses.

**Table 2:** Participant characteristics and unadjusted associations with interprofessional collaboration-IPC

		MH Primary Care Teams (PCTs) (*N* = 101)				MH Specialized Service Teams (SSTs) (*N* = 214)			
		Distribution		Bivariate analyses		Distribution		Bivariate analyses	
Blocks	Variables	n/Mean	%/SD	Beta*	P	n/Mean	%/SD	Beta*	P
Dependent variable	IPC (Mean/SD)	8.02	3.75			19.93	3.75		
1. Individual Characteristics	Age (Mean/SD)	41.71	10.56	−.299	.002	44.07	10.38	−.125	.069
	Gender (n/%)								
	Female	79	78.2			140	65.4		
	Male	22	21.8			74	34.6		
	Seniority in the team (Mean/SD)	2.25	3.07			3.44	5.56		
	Belief in the benefits of IPC score^a^ (Mean/SD)	5.99	0.82	.445	< .001	6.35	.65	.238	< .001
2. Interactional Characteristics	Knowledge sharing score^a^ (Mean/SD)	5.51	0.97	.556	< .001	5.83	.85	.330	< .001
	Knowledge integration score^a^ (Mean/SD)	3.91	0.99	.709	< .001	4.41	1.16	.609	< .001
	Affective commitment toward the team score^a^ (Mean/SD)	4.43	1.19	.462	< .001	5.06	1.20	.470	< .001
	Participation in decision-making score^a^ (Mean/SD)	4.44	1.45	.455	< .001	5.28	1.24	.408	< .001
	Mutual trust score^a^	5.13	1.05	.204	.004	5.23	1.21	.519	< .001
	Team climate score^b^ (Mean/SD)	19.45	3.03	.676	< .001	20.92	3.48	.686	< .001
	Team conflict score^a^ (Mean/SD)	3.39	1.79	−.128	.202	9.23	3.29	−.356	< .001
	Team autonomy score^a^ (Mean/SD)	4.48	1.29	.162	.0106	5.12	1.12	0322	< .001
3. Structural Characteristics	Team size (Mean/SD)	7.02	2.32			8.44	3.85		
	Organizational Support score^b^ (Mean/SD)	4.61	1.23	.342	< .001	4.95	1.13	.0449	< .001
4. Professional Role Characteristics	Type of profession (n/%)								
	Medical professions	16	15.8			93	43.5		
	Psychosocial professions	77	76.2			95	44.4		
	General Professions	8	7.9			26	12.1		
	Multifocal identification score^a^ (Mean/SD)	20.22	2.54	.517	< .001	20.97	2.61	.461	< .001

^a^ Mean score (1 to 7 for each variable); min: 1, max: 7; higher = positive ^b^ Mean score (1 to 7 for 4 dimensions); min: 4, max: 28; lower = positive

Beta*: Standardized coefficients Beta

The multiple linear regression models are presented in Table 3. For MH-PCTs, four variables were independently and positively associated with IPC, including three related to Interactional characteristics (knowledge sharing, knowledge integration and team climate), and one related to Professional role characteristics (multifocal identification). This model explained 63.6% of total variance (adjusted R^2^), and had acceptable goodness-of-fit. In the MH-SSTs, four variables were independently and positively associated with IPC, of which two related to Interactional characteristics (knowledge integration and team climate), one related to Structural characteristics (organizational support), and another related to Professional role characteristics (multifocal identification). The single variable related to Individual characteristics, age, was significantly and negatively associated with IPC. This model explained 55.0% of total variance (adjusted R^2^), and had acceptable goodness-of-fit.

**Table 3:** Variables independently associated with interprofessional collaboration (IPC): Multiple linear regressions

Linear regression models		Standardized Coefficients Beta	t	Sig.	95.0% Confidence Interval for B		Collinearity Statistics	
					LB	UB	Tolerance	VIF
MH Primary Care Teams (PCTs)^a^								
Total sample (N = 101)	(Constant)		−1.584	.116	−7.259	.814		
Interactional Characteristics	Knowledge sharing score	.238	3.463	.0001	.395	1.457	.767	1.303
	Knowledge integration score	.375	4.547	< .001	.796	2.030	.536	1.866
	Team climate score	.265	3.119	.002	.119	.537	.504	1.984
Professional Role Characteristics	Multifocal identification score	.141	1.975	.005	−.001	.419	.710	1.408
MH Specialized Service Teams (SSTs)^b^								
Total sample (N = 214)	(Constant)		1.776	.077	−.341	6.536		
Individual Characteristics	Age (Mean/SD)	−.114	−2.450	.015	−.074	−.008	.973	1.028
Interactional Characteristics	Knowledge integration score	.240	3.819	<.001	.376	1.179	.535	1.871
	Mutual trust score	.124	1.852	.065	−.025	.795	.469	2.131
	Team climate score	.323	3.828	<.001	.169	.528	.297	3.371
	Team autonomy score	.084	1.699	.091	−.042	.567	.868	1.152
Structural Characteristics	Organizational Support score	.106	1.931	.005	−.007	.709	.704	1.420
Professional Role Characteristics	Multifocal identification score	.094	1.689	.005	−.023	.293	.682	1.467

MH Primary Care Teams^a^: Adjusted R^2^: 0,636; Goodness of fit–F: 44.696 *P* < 0.001

MH Specialized Service Teams^b^: Adjusted R^2^: 0.550; Goodness of fit–F: 38.159 *P* < 0.001

## Discussion

This study was to our knowledge the first to compare variables associated with IPC in MH-PCTs and MH-SSTs. The findings revealed three variables independently and positively associated with IPC in both PCTs and SSTs (knowledge integration, team climate and multifocal identification); whereas knowledge sharing correlated with MH-PCTs only, and two other variables (organizational support and age) with MH-SSTs only. Therefore, in terms of variables significantly associated with ICP in MH-PCTs and MH-SSTs, there were as many differences as similarities, contrary to our hypothesis. This may be explained by the differences in teams working in primary care as compared with specialized care, described in the introduction, and by the fact that most PCTs were formed following the MH reform with staff from former SSTs. The increased promotion of integrated care among professionals from both PCTs and SSTs aimed at accommodating patients who need service at both levels of care may also explain our results. Moreover, while the proportion of medical professionals was greater among SSTs, and psychosocial professionals more prevalent among MH-PCTs, inter-professional collaboration (IPC) was not associated with type of professional in either MH-PCTs or MH-SSTs.

In addition, all four blocks in the conceptual framework were represented for MH-SSTs, whereas only two blocks represented MH-PCTs. No Individual or Structural characteristics variables were associated with IPC in MH-PCTs. This result may be explained by the recent nature of professional transfers to the new multidisciplinary MH-PCTs, and the early stage of team operations in terms of staff acquisition, the transfer of task-related knowledge, skill development as well as team support [68]. The embedding of IPC competencies (communication, synchronization, explicit coordination and implicit coordination), and interdisciplinary values and skills as well as team support into team structures would be expected to improve over time. It is only over a long-period of time that IPC may become effective, resulting from practice-based training [69] and team building activities [70, 71] that would allow health professionals to break with old habits [70] by acquiring new knowledge, skills and attitudes [72]. By contrast, teamwork and other forms of collaboration were more the norm among psychiatrists and psychosocial professionals in specialized services, where cases are more complex and patient medical and social needs more recurrent.

Two variables related to Interactional characteristics (knowledge integration and team climate) were associated with IPC in both MH-PCTs and MH-SSTs. A high degree of knowledge integration and positive work environment are particularly important in managing most common MH problems, but also in treating severe and enduring MH problems such as schizophrenia and bipolar disorder. Studies have shown that IPC would be limited without knowledge integration from different disciplines and a positive working environment [30, 41]; while unsatisfactory or difficult conditions threaten the quality of patient care [53] and professional life [73, 74]. Knowledge integration and positive team climate may include better role clarification, inter-professional communication, and collaborative leadership, and this aligned with a patient-centred care approach based on a high level of IPC focused on the needs of patients and their loved-ones for improving quality of care [53]. Knowledge integration in particular involves the development of a relational dynamic in which professionals influence each other in analyzing the situations encountered, and in articulating a shared vision and plan of action [53, 69, 74–76]. Knowledge integration coupled with positive and productive work environments may foster positive contacts among professionals in different disciplines that would enhance empathic responses while reducing anxiety levels among professionals [77].

The multifocal identification variable under Professional role characteristics was also associated with IPC in both MH-PCTs and MH-SSTs. This underlines the importance of professional identification for improving IPC in MH teams practicing from a multidisciplinary model of care, such as those responsible for expert assessments, case reviews and management of individuals with complex needs. Indeed, professional identification was experienced as a great source of satisfaction and motivation for workers whose professional skills were valued by other team members [78]. Moreover, strong team identity helped overcome multiple issues including those involving professional diversity or patient management [79, 80]. Research has shown that successful teamwork was enhanced when team identification was sufficiently strong to moderate individual professional identifications [9]. In addition, numerous studies show that certain intergroup contacts may lead to harm and discrimination, which would require that a set of actions be taken to address power differentials [77].

The knowledge sharing variable under Interactional characteristics was associated with IPC in MH-PCTs only. This suggests the particular importance of knowledge sharing in the newly formed MH-PCTs dealing with patients mainly affected by common mental disorders and substance use disorders as well as multiple biopsychosocial needs that require sharing of information, skills, and expertise [81, 82]. Consistent with our results, a number of studies demonstrated that collaboration rarely succeeds without knowledge sharing on teams [30, 83]. Knowledge sharing is also known to increase worker productivity and organizational performance [84, 85].

Organizational support, a Structural characteristics variable, was associated with IPC for MH-SSTs only. Organizational support entails multi-level leadership in overseeing the provision of team resources, adequate space for patient care, clear rules and procedures [2, 30]*,* a climate conducive to the development of good team working relationships [86], as well as leadership and administrative support [29]. This support is particularly essential in MH-SSTs where a variety of professionals treat cases involving complex and recurring disorders (e.g. schizophrenia, bipolar disorders), and some agitated and/or aggressive patients [87, 88]. Specialized care interventions also tend to occur in an emergency or crisis context, requiring a high level of organizational support. *O*rganizational support has also had positive effects for reducing staff turnover [64] and on job performance [51].

Regarding Individual characteristics, our study identified a negative association between age and IPC, unlike some previous research (e.g. [31]), but only in MH-SSTs. This finding may have been due to the prevalence of younger professionals working in MH-SSTs, whose relative lack of work experience may have encouraged greater collaboration with others as compared with more experienced team members in order to minimize errors [89]. Another explanation may be that younger MH professionals are more open to innovation, and to knowledge acquisition, as in the adoption of best practices and IPC. Moreover, since around 2012 all Canadian programs for health professional have been accredited to provide interprofessional education related to their standards [90], which may prepare health science students to work in collaboration prior to their entry into professional practice. However, despite a growing body of research, little consensus exists concerning the effects of age on IPC, as younger professionals may also work with seniors who manage to enforce the status quo [31, 91].

Finally, some independent variables identified as significant in other studies were not related to IPC in this research, whether for MH-PCTs or MH-SSTs, which may be partly explained by the study context. However, some variables that were not in collinearity with others, but measured different dimensions, may have been relatively close in meaning (e.g. multifocal identification and belief in benefits of interdisciplinary collaboration). Most astonishing was that type of profession (in Professional role characteristics) was not related to IPC in our study. This result may have occurred because teams were too similar in terms of their mix of professionals to be differentially related to IPC, or perhaps due to the way in which each individual team was regrouped in terms of PCTs or SSTs.

## Limitations

Despite some important findings, this study had certain limitations. First, the study used a cross-sectional design, which did not permit the formulation of cause and effect inferences about the data. Second, the research did not include a control group, which would have allowed for comparisons. Third, MH-PCTs and MH-SSTs represented a diversified range of teams such as assertive community treatment teams, emergency department teams (SSTs), single access point or psychosocial teams (PCTs), that were not treated as specific team types in the analyses. Treating each team subtype separately may have produced different IPC associated variables. Studying specific teams with a complementary method such as case study analysis may also have brought additional information concerning team processes and dynamics to the results. In addition, there were more SST than PCT teams, and for some, such as emergency department teams, response rates were relatively low. Fourth, some variables identified as key variables in earlier ICP research (e.g. leadership, team power balance, and team culture) were not investigated in the present study [3, 28]. Fifth, since Likert-scales of the French instruments used in this study often differed from their original version, the mean scores may not be compared with those from previous studies. Sixth, IPC could have been measured with other validated instruments than the one used in this study (e.g. Assessment of Interprofessional Team Collaboration Scale [92]). Seventh, multivariate linear regression analyses cannot identify IPC moderators or mediators. A study using equation modelling analysis could be a further step for identifying such IPC data. Finally, the results may only be generalized to the Quebec MH system, and to the sites included in this study.

## Conclusion

This study was innovative in a number of ways. First, it included a large sample of professionals working in MH-PCTs or MH-SSTs located in four Quebec MH networks. Second, the study tested numerous variables previously identified as strongly related to IPC and organized within a conceptual framework. Moreover, it identified variables associated with IPC in both MH-PCTs and MH-SSTs, comparing IPC associated variables for the two practice settings. The two multivariate regression models identified three independent variables related to Interactional characteristics, and one each for Individual characteristics, Structural characteristics, and Professional role characteristics, respectively. Three independent variables were associated with both MH-PCTs and MH-SSTs, and three were specific to either MH-PCTs or MH-SSTs; as such, they reveal some variation in IPC across levels of care. This suggests the need for managers to promote the development and implementation of differentiated professional skills on teams, depending upon their required level of care provision. Surely, the maintenance of IPC depends upon the contributions of all team members; whereas their effectiveness may be directly influenced by managers themselves. Thus, at the level of specialized services, managers might focus their attention on organizational support without neglecting other variables identified in association with IPC in both MH-PCTs and MH-SSTs (i.e. knowledge integration, positive team climate and multifocal identification). At the primary MH level, managers should focus on the development of knowledge-sharing competencies. Finally, more outreach activities and training of MH professionals are needed to promote interdisciplinary values and skills as well as interprofessional knowledge and IPC. All in all, MH professionals working as members of multidisciplinary teams need preparation and support to know how to work more effectively and collaboratively.

## Data Availability

Signed confidentiality agreements prevent us from sharing the data. However, a copy of questionnaires may be obtained from the first author on request.

## Abbreviations

HSSCs: Health and social service centers
IPC: Interprofessional collaboration
LHSNs: Local health service networks
MH: Mental health
MH-PCTs: Primary mental health care teams
MH-SSTs: Specialized mental health service teams
PCTs: Primary care teams
SSTs: Specialized service teams
